# A New Water-Soluble Thermosensitive Star-Like Copolymer as a Promising Carrier of the Chemotherapeutic Drug Doxorubicin

**DOI:** 10.3390/ma14133517

**Published:** 2021-06-24

**Authors:** Mariia Chernykh, Dmytro Zavalny, Viktoriya Sokolova, Stanislav Ponomarenko, Svitlana Prylutska, Yuliia Kuziv, Vasyl Chumachenko, Andrii Marynin, Nataliya Kutsevol, Matthias Epple, Uwe Ritter, Jacek Piosik, Yuriy Prylutskyy

**Affiliations:** 1 Taras Shevchenko National University of Kyiv, Volodymyrska Street, 64, 01601 Kyiv, Ukraine; ergo.mari@gmail.com (M.C.); realaros@bigmir.net (D.Z.); stasponomarenko@ukr.net (S.P.); garaguts.yulia.fox@gmail.com (Y.K.); chumachenko_va@ukr.net (V.C.); kutsevol@ukr.net (N.K.); 2 Center for Nanointegration Duisburg-Essen (CeNIDE), Institute of Inorganic Chemistry, University of Duisburg-Essen, University Street, 5-7, 45117 Essen, Germany; viktoriya.sokolova@uni-due.de (V.S.); matthias.epple@uni-due.de (M.E.); 3 Department of Physiology, Plant Biochemistry and Bioenergetics, National University of Life and Environmental Science of Ukraine, Heroiv Oborony Street, 15, 03041 Kyiv, Ukraine; psvit_1977@ukr.net; 4 National University of Food Technologies of Ukraine, Volodymyrska Street, 01033 Kyiv, Ukraine; andrii_marynin@ukr.net; 5 Institute of Chemistry and Biotechnology, Technical University of Ilmenau, Weimarer Street, 25, 98693 Ilmenau, Germany; uwe.ritter@tu-ilmenau.de; 6 Laboratory of Biophysics, Intercollegiate Faculty of Biotechnology UG-MUG, University of Gdansk, Abrahama 58, 80-307 Gdańsk, Poland

**Keywords:** thermosensitive star-like copolymer, doxorubicin, HeLa cells, cytotoxicity, in vitro uptake

## Abstract

A new water-soluble thermosensitive star-like copolymer, dextran-graft-poly-N-iso-propilacrylamide (D-g-PNIPAM), was created and characterized by various techniques (size-exclusion chromatography, differential scanning calorimetry, Fourier-transform infrared (FTIR) spectroscopy, and dynamic light scattering (DLS) spectroscopy). The viability of cancer cell lines (human transformed cervix epithelial cells, HeLa) as a model for cancer cells was studied using MTT and Live/Dead assays after incubation with a D-g-PNIPAM copolymer as a carrier for the drug doxorubicin (Dox) as well as a D-g-PNIPAM + Dox mixture as a function of the concentration. FTIR spectroscopy clearly indicated the complex formation of Dox with the D-g-PNIPAM copolymer. The size distribution of particles in Hank’s solution was determined by the DLS technique at different temperatures. The in vitro uptake of the studied D-g-PNIPAM + Dox nanoparticles into cancer cells was demonstrated by confocal laser scanning microscopy. It was found that D-g-PNIPAM + Dox nanoparticles in contrast to Dox alone showed higher toxicity toward cancer cells. All of the aforementioned facts indicate a possibility of further preclinical studies of the water-soluble D-g-PNIPAM particles’ behavior in animal tumor models in vivo as promising carriers of anticancer agents.

## 1. Introduction

Cancer is the second leading cause of death worldwide after cardiovascular diseases. Nowadays, chemotherapy is the most common therapeutic method for treating cancer by the application of small toxic molecules that interact with DNA and cause cell death. However, these chemotherapeutic drugs are nonselective and usually damage both cancer and healthy cells and have a short circulation half-life as well as limited targeting effects. Thus, regular chemotherapy leads to numerous side effects, including immediate toxicity and chronic toxicity [1], which can damage all human organs, including the heart, lungs, brain, etc. Modern technologies based on different nanoscale drug carriers [2,3,4,5], which help to minimize the negative side effects of chemotherapy on healthy cells, are being widely tested. Many synthetic polymers can be used as nanocarriers for intelligent drug delivery to tumor cells [6]. Such polymeric nanocarriers allow the carrying of highly toxic drugs specifically to cancer tissue and can control drug release due to the unique physical–chemical properties of polymer molecules. In recent years, a new generation of polymer-based nanocarriers based on smart pH- and redox-stimuli-responsive nanosystems have been reported [7]. Their specific chemical functionalities improve the drug loading and release, as well as cellular interactions.

Linear polymers are mostly used for drug delivery to specific cells and tissues [8]. Recently provided studies, however, demonstrated the high efficiency of a branched copolymer of dextran-polyacrylamide, both as a nanocarrier for drug delivery and as a matrix for the preparation of new nanosystems for antitumor photodynamic therapy [9,10,11]. Branched copolymers have a controlled molecular structure and high local concentration of functional groups, which make them promising objects for further biomedical technologies. Moreover, it is possible to use such smart polymers in oncology, especially for the encapsulation of highly toxic drugs as well as accurate targeting and controlled release [12,13].

Doxorubicin (Dox) is a well-known chemotherapeutic drug used for anticancer therapy due to its high toxicity (the gold standard of chemotherapy). There are two proposed mechanisms by which Dox acts in the cancer cells: (i) intercalation into DNA and disruption of topoisomerase-II-mediated DNA repair and (ii) generation of free radicals and their damage to cellular membranes, DNA, and proteins [14]. As it was reported [14], Dox is oxidized to semiquinone, an unstable metabolite, which is converted back to Dox in a process that releases reactive oxygen species (ROS). ROS can lead to lipid peroxidation and membrane damage, DNA damage, and oxidative stress and also trigger apoptotic pathways of cancer cell death [15].

Considering the above, this study focused on the creation and in vitro testing of the anticancer effect of a nanosystem based on a thermosensitive star-like copolymer, dextran-graft-poly-N-iso-propylacrylamide (D-g-PNIPAM), loaded via Dox to compare its efficacy toward the free drug. The targeted delivery of the highly toxic drug was the main aim of this research.

## 2. Materials and Methods

### 2.1. Material Preparation

Dox (Sigma-Aldrich, Co, Ltd., St. Louis, MO, USA) was used without further purification. The substance was dissolved in phosphate-buffered saline (PBS: 8.0 g NaCl, 0.2 g KCl, 1.44 g Na_2_HPO_4_ and 0.24 g KH_2_PO_4_ in 1 L of distilled water, pH = 7.4) with a maximum concentration of 20 µg mL^−1^.

A star-like copolymer, D-g-PNIPAM, was used as a carrier for Dox molecules. D-g-PNIPAM was synthesized via the radical graft polymerization method using a Ce(IV)/HNO_3_ redox system. PNIPAM was grafted on certified dextran with molecular weights M_w_ = 7 × 10^4^ g mol^−1^ (produced by Serva, UK). The number of grafts was controlled by the molar ratio of the added initiator to dextran to obtain a copolymer with 15 theoretical grafts. The synthesis and identification of the sample were described in detail in [16].

The molecular parameters of the D-g-PNIPAM copolymer used in this study are shown in Table 1.

For the present study, the stock solution of D-g-PNIPAM copolymer was prepared in distilled water (500 µg mL^−1^). The copolymer was then diluted by Hank’s solution. The D-g-PNIPAM copolymer and Dox were mixed in the corresponding volume ratios for obtaining water-soluble D-g-PNIPAM + Dox particles, namely: 125 + 5, 62.5 + 2.5, and 25 + 1 µg mL^−1^. All manipulations were performed at room temperature. 

### 2.2. Size-Exclusion Chromatography

Multidetection SEC using an SEC line of the Institut Charles Sadron (ICS, France) allowed us to determine the average molecular weights of the branched PNIPAM sample. This SEC line consists of a refractometer and two angles (7° and 90°) light scattering apparatus. The fractionation was performed through three columns (PLgel Mixed B) with a precolumn arranged in series. The eluent was N-methyl-2-pyrrolidone (NMP) of HPLC grade with 0.1 M LiBr. Measurements were performed at 60 °C using a constant flow rate of 0.5 mL min^−1^. A PNIPAM solution of concentration 3.33 g L^−1^ was filtered on a 0.45 mm membrane prior to being injected. A volume of 100 mL of the solution was injected.

### 2.3. Differential Scanning Calorimetry

The thermograms for D-g-PNIPAM in an aqueous solution were recorded with a SETARAM III microcalorimeter. The measuring cell was filled with a solution of D-g-PNIPAM (200 mg). The reference cell was filled with distilled water to the same mass as the first cell to within 0.1 mg. The lower critical solution temperature (LCST) was registered during the first cycle of heating at 1 °C min^−1^.

### 2.4. Fourier-Transform Infrared Study

Fourier-transform infrared (FTIR) spectroscopy was used to study the interaction of the D-g-PNIPAM copolymer with Dox molecules. FTIR spectra were obtained on a Nicolet NEXUS-475 (Waltham, MA, USA) spectrophotometer in the range 4000–400 cm^−1^ using thin films with thicknesses of 6–9 µm. The films were cast from the aqueous solution of D-g-PNIPAM with Dox as well as without any adding. 

### 2.5. Dynamic Light Scattering 

The size distribution for studied systems in Hank’s solution was determined via the dynamic light scattering (DLS) method on a Zetasizer Nano-ZS90 (Malvern, Worcestershire, UK) at different temperatures. The instrument was equipped with a He–Ne laser (5 mW) operating at a wavelength of 633 nm. The autocorrelation function of the scattered light intensity was analyzed by the Malvern Zetasizer software 7.12 with the Smoluchowski approximation.

### 2.6. Cell Culture

The HeLa cells (American Type Culture Collection (ATCC CCL-2), Rockville, Maryland, MD, USA) were cultured in Dulbecco’s modified eagle medium (DMEM), supplemented with 10% fetal calf serum (FCS), 100 U mL^−1^ penicillin, and 100 mg mL^−1^ streptomycin at 37 °C in a humidified atmosphere with 5% CO_2_. Twelve hours before the uptake experiments, the cells were trypsinized and seeded in 48-well plates with 25 × 10^3^ cells per well in 0.25 mL DMEM with FCS for studied cells.

HEK293 (human embryonic kidney) cells were seeded in 96-well plates with 10 × 10^3^ cells per well in DMEM supplemented with 10% FCS, 50 U mL^−1^ penicillin, and 100 µg mL^−1^ streptomycin at 37 °C in a humidified atmosphere with 5% CO_2_. 

### 2.7. MTT Assay

The HeLa cells’ viability was analyzed via the MTT (3-(4.5-dimethylthiazol-2-yl)-2.5-diphenyl-tetrazolium bromide; Sigma, Taufkirchen, Germany) assay after 24 h of incubation with the studied particles. The final concentration of particles per well after dilution in the medium was (total volume of 250 µL per well): Dox: 5, 2.5, and 1 µg mL^−1^; D-g-PNIPAM copolymer: 125, 62.5, and 25 µg mL^−1^; D-g-PNIPAM + Dox: 125 + 5, 62.5 + 2.5, and 25 + 1 µg mL^−1^. In this case, we took into account the value of IC_50_ for Dox in HeLa cells after 24 h of incubation as 2.2 µg mL^−1^ [17]. 

The HEK293 cells’ viability was analyzed via the MTT assay after 24 h of incubation with D-g-PNIPAM particles at different concentrations (125, 62.5, and 25 µg mL^−1^). 

MTT was dissolved in PBS (5 mg mL^−1^) and then diluted to 1 mg mL^−1^ in the cell culture medium. The cell culture medium of the incubated cells was replaced by 300 μL of the MTT solution. Cells were then incubated for 1 h at 37 °C under 5% CO_2_ in the humidified atmosphere. Three hundred microliters of dimethyl sulfoxide (DMSO) were added to the cells. After 30 min, a 100 μL aliquot was taken for spectrophotometric analysis with a Multiscan FC instrument (Thermo Fisher Scientific, Vantaa, Finland) at *λ* = 570 nm. The absorption of incubated cells was normalized to that of control (untreated) cells, thereby indicating the relative level of cell viability.

### 2.8. Live/Dead Assay

A Live/Dead assay was carried out according to the following protocol. Twenty-four hours after the incubation of HeLa cells with 62.5 μg mL^−1^ D-g-PNIPAM, 2.5 μg mL^−1^ Dox, and D-g-PNIPAM + Dox (62.5 + 2.5 μg mL^−1^) particles, cells were washed with PBS and stained with a Live/Dead viability/cytotoxicity assay for mammalian cells (L3224, Invitrogen Co., Waltham, MA, USA) to evaluate the cell viability. One hundred and fifty microliters of a calcein AM and ethidium homodimer-1 working solution were directly added to these cells. Afterward, cells were subsequently incubated for 30 min at 37 °C. The Live/Dead kit determines the cell viability based on the cell membrane integrity. Living cells are stained by calcein AM, which emits green fluorescence (517 nm) when excited by blue light (494 nm), whereas dead cells are stained by EthD-1, which emits red fluorescence (617 nm) when excited by green light (528 nm). 

The cell viability (Live/Dead assay) was determined via fluorescence microscopy. A Keyence Biorevo BZ-9000 instrument (Osaka, Japan), equipped with filters for FITC and TRITC with 10× and 20× objectives, was used. All images were recorded with the BZ-II viewer software and further processed with the BZ-II analyzer software.

### 2.9. Confocal Laser Scanning Microscopy 

The uptake of particles present in the samples was observed as follows. Thirty microliters of the fluid was added to the cells. The HeLa cells were incubated with particles for 24 h. Immediately after the indicated time, cells were washed three times with PBS in order to remove all dispersed particles, fixed with 4% paraformaldehyde at room temperature for 10 min, then stained with fluorescent dye DAPI for the cell nucleus and washed three times with PBS. Finally, cells were studied via confocal laser scanning microscopy (CLSM), which provided the information on whether particles were able to enter these cells. CLSM was performed on a TCS SP8 AOBS system controlled by the LAS X software 3.0 (Leica Microsystems, Wetzlar, Germany). The laser lines used for excitation were DPSS 561 nm (for Dox) and Diode 405 nm (DAPI). Images were acquired with a 20× objective. 

### 2.10. Statistics

All experiments were carried out in triplicate. Statistical analysis of the data was performed using one-way analysis of variance (ANOVA) with Tukey’s post hoc test or Student’s t-test (OriginLab Corporation, Northampton, MA, USA). The difference was considered statistically significant at *p* < 0.05.

## 3. Results

### 3.1. Material Characterization

**Figure 1:**
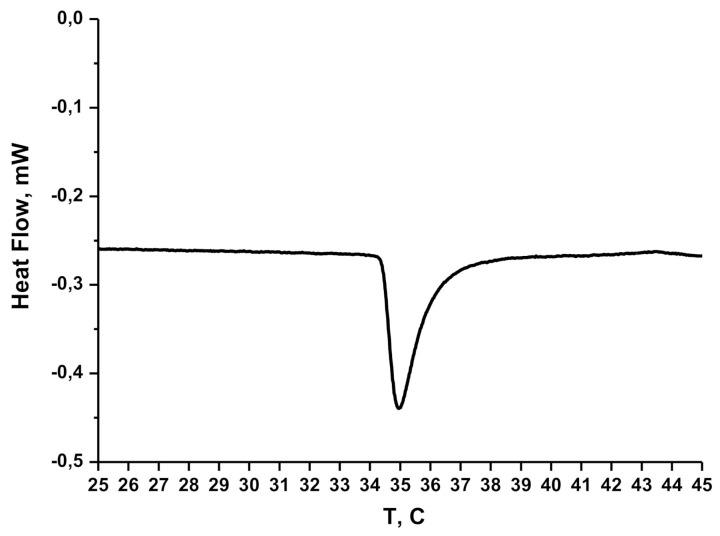
Micro DSC thermogram for D-g-PNIPAM in aqueous solution (CD-g-PNIPAM = 125 µg mL^−1^).

We controlled the region of the polymer carriers’ phase transition via micro differential scanning calorimetry (DSC). Figure 1 demonstrates that the exothermic phase transition started at 34 °C and completely finished at 37 °C. 

**Figure 2:**
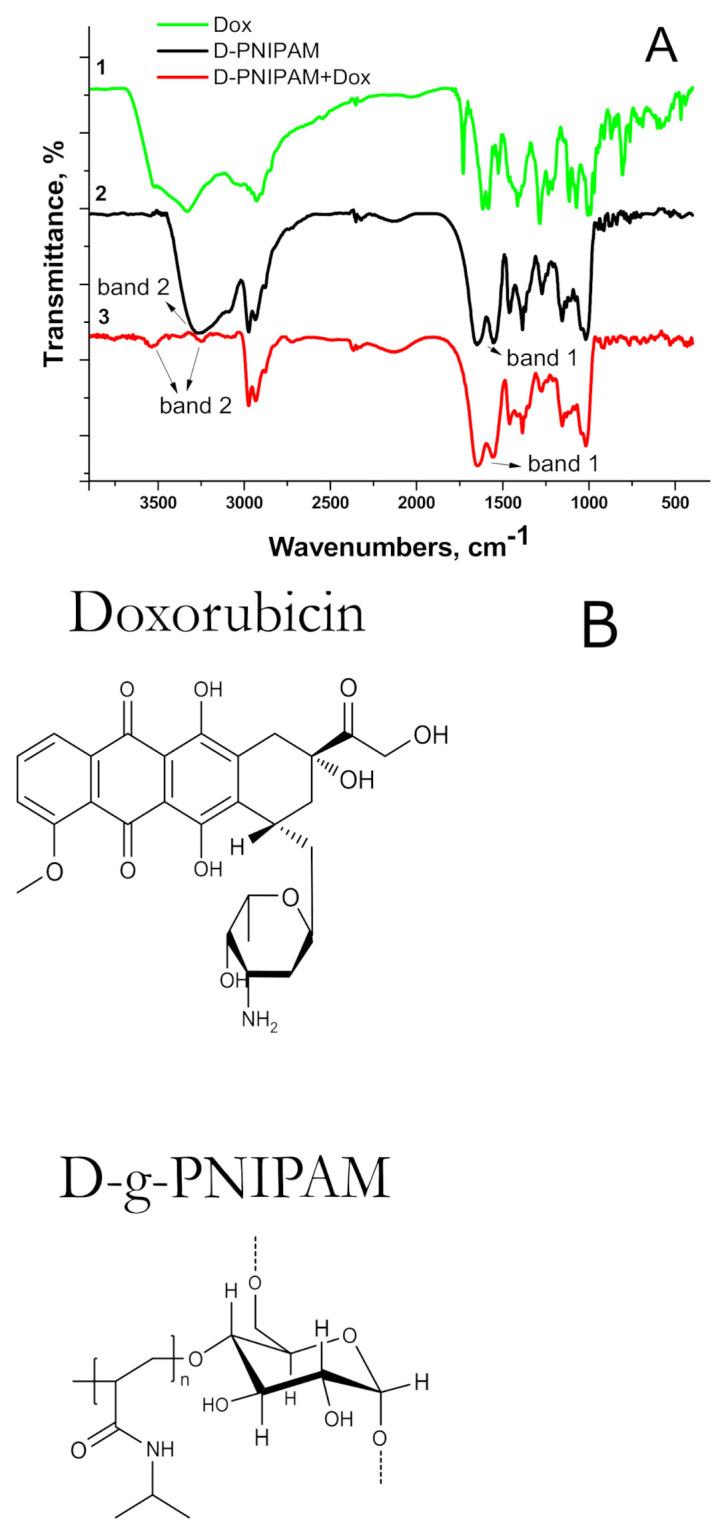
(**A**) FTIR spectra for: 1—Dox (5 µg mL^−1^), 2—D-g-PNIPAM (125 µg mL^−1^), and 3—D-g-PNIPAM + Dox (125 + 5 µg mL^−1^) complex; (**B**) chemical structures of Dox and D-g-PNIPAM.

The FTIR spectra of the D-g-PNIPAM, D-g-PNIPAM + Dox as well as individual Dox are shown in Figure 2.

The characteristic bands of Amide 1 were clearly expressed in the spectrum of the individual copolymer (1650–1660 cm^−1^, C=O) and Amide 2 (1615 cm^−1^, N-H) (Figure 2, band 1). The FTIR spectrum of Dox was similar to that obtained in [18]. A wide band in the region of 3500 cm^−1^ was observed for all samples. The measurements were performed for films that were cast from an aqueous solution. Since the copolymer is hydrophilic, it retains up to 10% water even when dried. When the D-g-PNIPAM + Dox complexes were formed, the hydrophilic functional groups of macromolecules bound the small molecules of drugs, which led to alteration of the hydrophilicity of the systems, explaining the slight difference in the bands of the copolymer or complex in the range of 3500–2900 cm^−1^ (Figure 2, band 2). These bands are a superimposition of a wide band of oscillations of -OH water groups. 

For the D-g-PNIPAM + Dox system, the disappearance of the absorption band at 1730 cm^−1^ corresponding to C=O Dox groups was observed. In addition, some changes in the characteristics of bands Amide 1 and Amide 2 were registered in comparison with individual D-g-PNIPAM copolymers. These changes were more expressed for NH_2_ groups in the D-g-PNIPAM + Dox complex. Thus, the FTIR study testified to the interaction between the copolymer and Dox.

**Figure 3:**
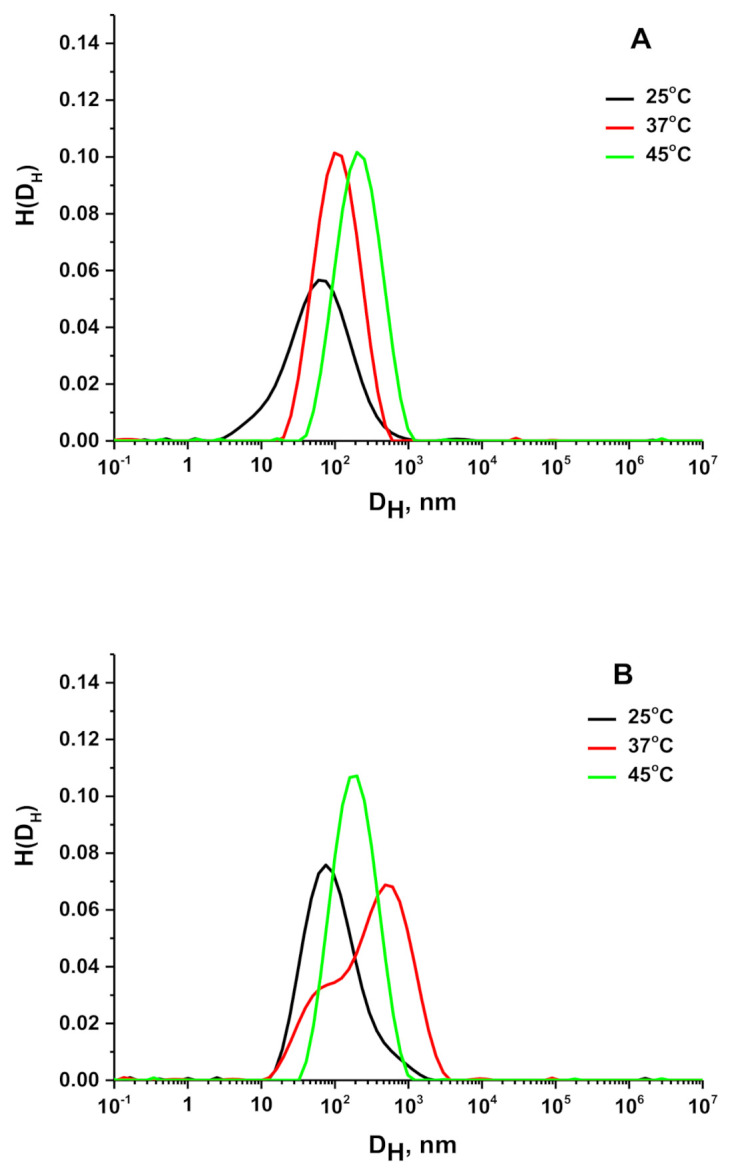
Hydrodynamic diameter distributions for D-g-PNIPAM (**A**) and D-g-PNIPAM + Dox system (**B**) at different temperatures.

Figure 3A and Table 2 represent the change in the size of the scattering objects for the D-g-PNIPAM in Hank’s solution at different temperatures. We need to emphasize that for D-g-PNIPAM the DLS was performed in the Guiner regime. At 25 °C, D-g-PNIPAM macromolecules were around 60 nm in size. At 37 and 45 °C, the size of scattering D-g-PNIPAM objects increased to 110 and 220 nm, respectively, and both peaks became narrower in comparison with the one at 25 °C. 

Figure 3B represents the behavior of the D-g-PNIPAM + Dox system in Hank’s solution at 25, 37, and 45 °C. It can be seen that at 25 °C the size of scattering objects was slightly bigger in comparison with individual D-g-PNIPAM in Hank’s solution (Table 2). In Hank’s solution, D-g-PNIPAM macromolecules underwent aggregation when the temperature increased from 25 to 37 °C, exceeding the LCST value (Figure 3B). It should be noted that a two-peak size distribution had been observed for the D-g-PNIPAM + Dox system at 37 °C. This indicates the beginning of the aggregation process. A further temperature increase to 45 °C caused the finishing of the aggregates formation (Table 2). Finally, the particle size distribution can be also derived from the polydispersity index (PDI) value [19]. The registered PDI values > 0.5 indicate a highly polydisperse distribution of the tested samples (Table 2).

### 3.2. In Vitro Cell Culture Study

**Figure 4:**
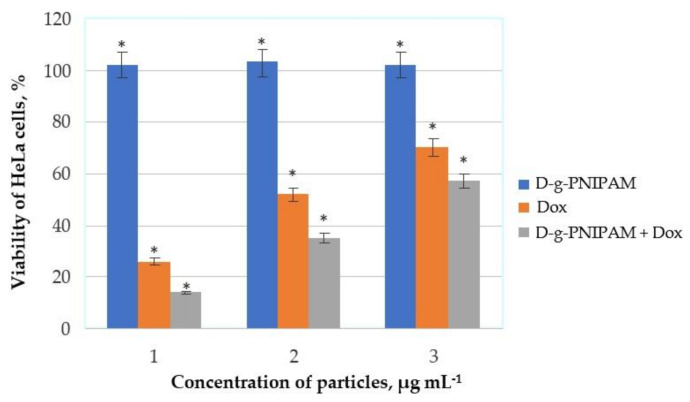
MTT assay results on HeLa cells after 24 h of incubation with D-g-PNIPAM, Dox, and D-g-PNIPAM + Dox particles at different concentrations: 125, 5 and, 125 + 5 µg mL^−1^ (1); 62.5, 2.5, and 62.5 + 2.5 µg mL^−1^ (2); 25, 1, and 25 + 1 µg mL^−1^ (3). Data are given relative to the untreated control samples (* *p* < 0.05).

The MTT assay (Figure 4) showed that D-g-PNIPAM particles were not toxic for the HeLa cells for 24 h. Moreover, they did not show any toxic effect against the noncancer HEK293 cells used for comparison (data not shown). With a change in concentration in the range 1–5 µg mL^−1^, Dox killed about 30–74% of HeLa cells and D-g-PNIPAM + Dox about 43–86%, respectively.

**Figure 5:**
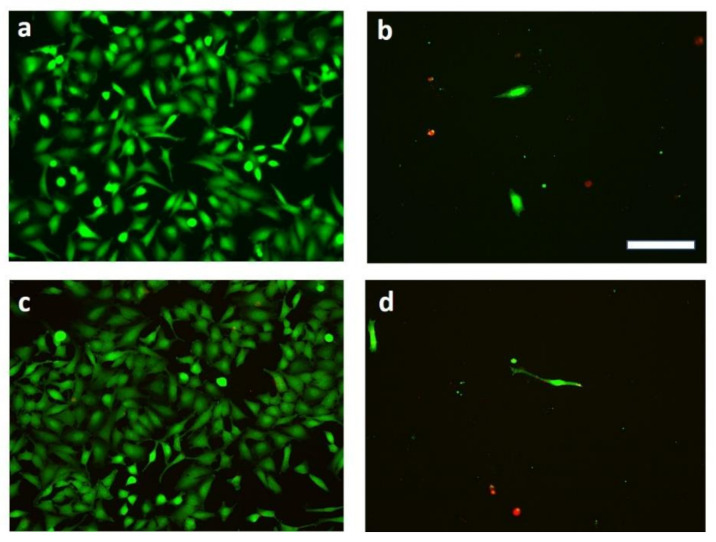
Representative Live/Dead staining of HeLa cells after 24 h of incubation with (**a**) D-g-PNIPAM (62.5 μg mL^−1^); (**b**) Dox (2.5 μg mL^−1^); (**c**) mock (untreated cells); (**d**) D-g-PNIPAM + Dox (62.5 + 2.5 μg mL^−1^). Green cells are living cells and red ones are dead. Scale bar 20 µm (for all images).

The Live/Dead assay (Figure 5) showed that free Dox and its composition with D-g-PNIPAM did not allow HeLa cells to divide after 24 h of incubation.

**Figure 6:**
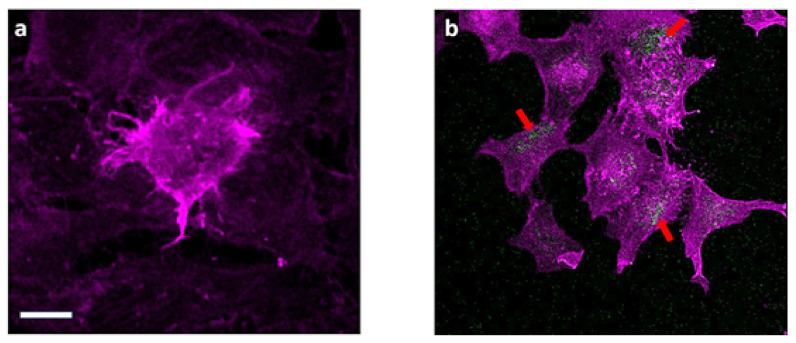
CLSM images of: (**a**) mock (untreated cells); (**b**) HeLa cells after 24 h of incubation with D-g-PNIPAM + Dox (25 + 1 μg mL^−1^). Arrows indicate the accumulation of nanoparticles inside cancer cells. actin staining (magenta), and particles (green). Scale bar 10 μm (for both images).

The cellular uptake and the distribution of D-g-PNIPAM + Dox after 24 h of incubation on HeLa cells is presented in Figure 6. It should be underlined that particles were precisely washed in all these cases to remove adhering or dispersed residues.

## 4. Discussion

The choice of this star-like copolymer was based on the growing need for novel intelligent biocompatible polymers. Thermally responsible polymers based on poly(N-isopropylacrylamide) (PNIPAM) constitute a promising approach for the creation of materials for biomedical applications [20]. PNIPAM exhibits an LCST value, below which the polymers are soluble [21]. Above the LCST, this polymer undergoes a phase transition, then collapses and forms aggregates. Linear PNIPAM has an LCST value of approximately 32 °C. Below 32 °C, it is hydrophilic and water-soluble; above 32 °C, it is partially hydrophobic. Thus, PNIPAM is soluble at room temperature and undergoes conformational transition at a physiological temperature (32 °C), therefore it can be considered interesting for biomedical application, especially for encapsulation of hydrophilic drugs at room temperature with their further controlled release at physiological temperatures (around 37 °C). The shift of the phase transition region to a temperature closer to 37 °C would be a real achievement in pharmaceutical materials. It was demonstrated [16] that for the D-g-PNIPAM copolymer used in this study the temperature of transition is higher than the LCST point for a linear PNIPAM of similar molecular weight and polydispersity [22]. The region of the LCST was studied by using the DLS technique. It showed a drastic change in the shape of size distribution curves and a scattering intensity decrease at 32–34 °C. Such change resulted in a conformational transition of D-g-PNIPAM macromolecules, which become partially hydrophobic. The aggregation process was then observed until 36–37 °C. Further heating did not cause additional aggregation. A micro-DSC thermogram for D-g-PNIPAM in aqueous solution (Figure 1) clearly demonstrated that the exothermic phase transition started at 34 °C and completely finished at 37 °C. Moreover, we showed earlier [23,24] that branched PNIPAM-based polymers, due to their more compact molecular structure, have a higher local concentration of functional groups in comparison to their linear analogs. Thus, D-g-PNIPAM can be used for encapsulation of hydrophilic Dox molecules with their further release after conformation transition of the polymer carrier when it becomes partially hydrophobic. The FTIR spectral changes for the D-g-PNIPAM + Dox binary system in comparison with the individual components (D-g-PNIPAM and Dox) indicated the interaction of the polymer and the antitumor drug (Figure 2).

The biological testing required the preparation of polymer systems in a buffer solution. We used Hank’s balanced salt solution for the dilution of the prepared stock polymer solution. It should be noted that Hank’s solution can provoke an additional aggregation process in the polymer systems in the region of the LCST value, which can negatively affect their biological efficacy. That is why the DLS study of the D-g-PNIPAM and D-g-PNIPAM + Dox system prepared in Hank’s solution was performed to control this process in the region of the LCST value of the polymer carrier. The experiment was carried out at 25, 37, and 45 °C.

At room temperature (25 °C) D-g-PNIPAM macromolecules were around 60 nm in size (Figure 3A and Table 2). Obviously, this polymer is not a polyelectrolyte, that is why the additional salt balanced solution would not affect drastically the size and conformational changes of D-g-PNIPAM macromolecules. However, in the salt solution, the hydrophilic–hydrophobic balance of polymer macromolecules can slightly change, as the hydrophilic functional groups are blocked by components of Hank’s solution. That can lead to the shrinking of the polymer macromolecules and can cause the formation of some aggregates. At 37 and 45 °C, the size of scattering D-g-PNIPAM objects increased to 110 and 220 nm, respectively. Such behavior can be explained by increased aggregation processes after the LCST value of the polymer.

In the presence of Dox, two types of scatters were observed (Figure 3B): the second term is the shoulder on the right side of the main peak corresponding to the fraction of the aggregates. Probably, polyfunctional Dox molecules caused partial aggregation of D-g-PNIPAM macromolecules. At 37 °C, the hydrodynamic diameter of solution components did not change drastically. However, the intensity of the second peak increased significantly. It could be explained by the aggregation of D-g-PNIPAM macromolecules in a solution above the LCST value. At 45 °C, only a single peak was observed demonstrating the completion of macromolecular shrinking.

The MTT and Live/Dead assay results showed it may be possible to increase the dose of the drug and maintain cell viability. The MTT tests did not show any toxic effect of D-g-PNIPAM particles against the normal (noncancer) HEK293 cells as well as HeLa cells for 24 h (Figure 4). On the contrary, the effectiveness of D-g-PNIPAM + Dox nanoparticles cytotoxic action increased with growing Dox content by an average of 1.5 times compared to the free Dox action. Finally, the Live/Dead assays showed that free Dox and its composition with D-g-PNIPAM did not allow cancer cells to divide (Figure 5).

The membranotropic effect of D-g-PNIPAM particles on cancer cells was studied via the CLSM technique with the usage of Dox as a fluorescent label [25,26]. The cellular uptake and the distribution of D-g-PNIPAM + Dox nanoparticles after 24 h of incubation on HeLa cells is presented in Figure 6.

Thus, since the D-g-PNIPAM particles were not toxic for both cancer and normal cells, one can suggest applying them as targeted carriers for antitumor drugs such as Dox.

Importantly, the cardiotoxicity of Dox is one of the main factors responsible for the limitation of Dox use in oncology. The emergence of such cardiotoxic effects is dose-dependent and sharply increases with high cumulative doses of the drug, resulting in a decrease in the antitumor activity of Dox. Therefore, the inclusion of Dox into the D-g-PNIPAM structure stabilizes the anticancer activity of the drug. Additionally, it allows a significant increase in the target dose of Dox. Moreover, a water-soluble D-g-PNIPAM copolymer is strongly sensitive to the temperature of the medium [16]. Therefore, it can be assumed that getting to the sites of pathology with elevated temperatures (~37 °C), D-g-PNIPAM particles undergo conformational rearrangement and, thus, release drugs locally. However, confirmation would require further in vivo testing.

## 5. Conclusions

To summarize, a novel water-soluble star-like copolymer-containing an anticancer drug, D-g-PNIPAM + Dox, was created and confirmed via SEC, DSC, DLS, and FTIR techniques. It was established that the D-g-PNIPAM + Dox nanoparticles decrease the viability of HeLa cells at low concentrations (1–5 µg mL^−1^) for 24 h in comparison to free Dox (up to 17%). The in vitro uptake of D-g-PNIPAM + Dox nanoparticles into cancer cells was shown via the CLSM method. The presented results showed that the D-g-PNIPAM copolymer is a promising platform for drug delivery and D-g-PNIPAM + Dox nanoparticles are also promising targets for further preclinical trials.

## Figures and Tables

**Table 1:** Molecular parameters of D-g-PNIPAM copolymer.

Sample	M_w_ × 10^−6^, g mol^−1^	M_n_ × 10^−6^, g mol^−1^	M_w_/M_n_
D-g-PNIPAM	1.03	0.674	1.52

**Table 2:** Evaluation of size change for D-g-PNIPAM and D-g-PNIPAM + Dox particles in Hank’s solution at different temperatures.

System	D_H_ (25 °C), nm	PDI (25 °C)	D_H_ (37 °C), nm	PDI (37 °C)	D_H_ (45 °C), nm	PDI (45 °C)
D-g-PNIPAM (125 µg mL^−1^)	60	0.75	110	0.52	220	0.56
D-g-PNIPAM + Dox (125 + 5 µg mL^−1^)	76	0.62	81 (additional peak at 515)	0.74	185	0.54

## Data Availability

Data Sharing is not applicable.
